# Sport-specific performance assessment with oxygen uptake measurements in time trials and critical power tests

**DOI:** 10.1038/s41598-025-09900-4

**Published:** 2025-07-21

**Authors:** Fabienne Bruggisser, Raphael Knaier, Raphael Schoch, Denis Infanger, Max Niemeyer, Arno Schmidt-Trucksäss, Jonathan Wagner

**Affiliations:** 1 Department of Sport, Exercise and Health, Faculty of Medicine, University of Basel, Basel, Switzerland; 2 Department Medicine, Training and Health, Institute of Sports Science and Motologie, Philipps-University Marburg, Marburg, Germany; 3 Department of Clinical Research, Faculty of Medicine, University of Basel, Basel, Switzerland

**Keywords:** Cardiopulmonary exercise tests, Oxygen consumption, Short duration exercise tests, Exercise physiology, Cardiopulmonary exercise testing, Physiology, Cardiovascular biology, Respiration

## Abstract

This study investigated the added value of peak oxygen uptake (${\dot{\text{V}}}$O_2peak_) measurements during a 4-min time trial (4TT) or a 3-min all-out test (3MT) as compared to a ramp cycling test to enhance sport-specific performance assessment. Data from two cross-sectional studies were analysed. In study 1, 18 male and 13 female recreational cyclists performed two ramp tests and two 4TT on different days. In study 2, 23 male and 17 female CrossFit athletes performed one ramp test followed by a 3MT with an intervening 20-min recovery. The tolerance limits for ∆${\dot{\text{V}}}$O_2peak_ between the two exercise tests was set to ± 0.13 L·min^−1^, reflecting common day-to-day variation in ${\dot{\text{V}}}$O_2peak_. In study 1, comparing the first ramp test with the first 4TT and comparing the second ramp test with the second 4TT showed mean differences of 0.02 L·min^−1^ (*p* = 0.930) and 0.03 L·min^−1^ (*p* = 0.873), respectively. However, the tolerance limits were − 0.35–0.31 L·min^−1^ and − 0.36–0.42 L·min^−1^, respectively. In study 2, the mean difference and tolerance limits were 0.08 L·min^−1^ (*p* = 0.637) and − 0.42–0.58 L·min^−1^. Although the 4TT and 3MT show limited agreement with ramp tests for ${\dot{\text{V}}}$O_2peak_, the lack of systematic deviations supports their value for sport-specific performance assessment.

**Supplementary Information:**

The online version contains supplementary material available at 10.1038/s41598-025-09900-4.

## Introduction

Cardiorespiratory fitness, measured as the highest oxygen uptake (${\dot{\text{V}}}$O_2peak_), determines the upper limit of the human oxygen transport and utilisation system^1–3^. Alongside the individual’s boundary delineating the transition between the heavy and severe intensity domains (determined by various markers including ventilatory and lactate thresholds or critical power) and work efficiency, the cardiorespiratory fitness is a key determinant in assessing endurance performance^4^. Hence, its measurement is routinely included in the physiological testing of athletes^5^.

Cardiopulmonary exercise testing (CPET) using incremental ramp protocols is currently the standard for quantifying cardiorespiratory fitness, specifically ${\dot{\text{V}}}$O_2peak_^1,2,6^. However, data measured using a ramp protocol provide limited insight into sport-specific performance parameters, such as anaerobic work capacity or pacing ability, as this protocol does not align with athletes’ typical training and competition regimens. In the context of competitive sport, the limited time available for testing necessitates optimising the extraction of data with minimal testing effort to effectively rank athletes, monitor physical characteristics and provide training prescriptions^7^. Previous studies have therefore suggested the use of more sport-specific exercise tests, such as time trials or critical power tests, as alternative methods to CPET to quantify ${\dot{\text{V}}}$O_2peak_^8–13^.

Time trials involve either completing as much work as possible in a given time or completing a given amount of work in a minimum amount of time^14^. A 4-min time trial (4TT) is a time-efficient and reliable performance test, highly correlated with longer efforts such as a 60-min time trial, elicits maximal aerobic performance near ${\dot{\text{V}}}$O_2peak_ at intensities approximating a 4-km individual pursuit, and shows high reproducibility with minimal variation in trained cyclists^14,15^. Further, critical power tests, such as the 3-min all-out test (3MT), are efficient and reliable, allowing for the evaluation of key physiological markers such as critical power, anaerobic work capacity and peak power output in a single session and significantly reducing the time and effort required compared to traditional methods involving multiple exhaustive trials on separate days^16,17^. Both the 4TT and 3MT are widely used to provide valuable insight into performance and benchmarking. Each test assesses specific physiological performance parameters that cannot be obtained from standardised ramp protocols. For example, the 4TT provides insight into pacing ability, aerobic capacity and heart rate^18^, while the 3MT explicitly measures anaerobic work capacity, peak power and fatigue index^16,19^.

Indeed, ${\dot{\text{V}}}$O_2peak_ achieved during a time trial and critical power test were found to be comparable to or even superior to ${\dot{\text{V}}}$O_2peak_ values achieved during a ramp test^8–13^. However, almost all studies concluding that time trials and all-out tests are feasible alternatives to ramp tests in assessing ${\dot{\text{V}}}$O_2peak_ compared group means/medians using *t*-test and correlation analyses^8,10,12,13^. *T*-test and/or correlation analyses are clearly not suitable for evaluating whether the results of a measurement method align with or validly reflect those of the gold standard method^20–22^. Those few studies that applied Bland–Altman analyses, however, did this without an a priori definition of an acceptable range, which is key for subsequent interpretation of the value of such time trial or critical power tests. Such a range could be defined based on different factors, as for example the expected day-to-day variation or measurement error^11^.

Integrating testing protocols that capture sport-specific performance with ${\dot{\text{V}}}$O_2peak_ measurements would improve the accuracy of performance assessment, increase the overall efficiency of the testing process, and not impose additional physical strain on the athlete. Therefore, this comparative study aimed to investigate the additional value of measuring oxygen uptake during a time trial and an all-out test in recreational cyclists and CrossFit athletes.

## Materials and methods

### Study design

This comparison used descriptive data from two cross-sectional studies conducted in the laboratories of the Department of Sport, Exercise and Health at the University of Basel, Switzerland. The two studies were conducted under consistent conditions (air humidity, 40–55%; room temperature, 20–22 °C) using equivalent devices between September 2020 and June 2021, and between December 2021 and December 2022. Both studies adhered to the Declaration of Helsinki and were approved by the Ethics Committee of Northwestern and Central Switzerland (EKNZ-2019-01697 and EKNZ-2021-00650). Written informed consent was obtained from all participants prior to the start of the studies.

Study 1 examined the usefulness of a self-paced 4TT to determine ${\dot{\text{V}}}$O_2peak_ in recreational cyclists. Participants completed four laboratory testing days, with a recovery phase of at least 24 h in between, and over a period of eight to ten days. On the first and third testing days, participants performed a CPET using a ramp protocol. On the second and fourth testing days, participants performed a 4TT. Study 2 examined the usefulness of a 3MT to determine ${\dot{\text{V}}}$O_2peak_ in highly trained CrossFit athletes. Participants attended one laboratory testing day only, which included a CPET using a ramp protocol, followed by a 3MT. As the participants had extensive experience of high-intensity exercise due to their sporting background, no familiarisation tests were carried out before the individual exercise tests. All tests were also preceded by standardised instructions to exert themselves until maximal voluntary exhaustion.

Combining both studies into a single manuscript allows for a more thorough evaluation of sport-specific performance assessment across different athletic populations, thereby enhancing the relevance of our findings for practitioners in a variety of sporting contexts.

### Participants

**Fig. 1:**
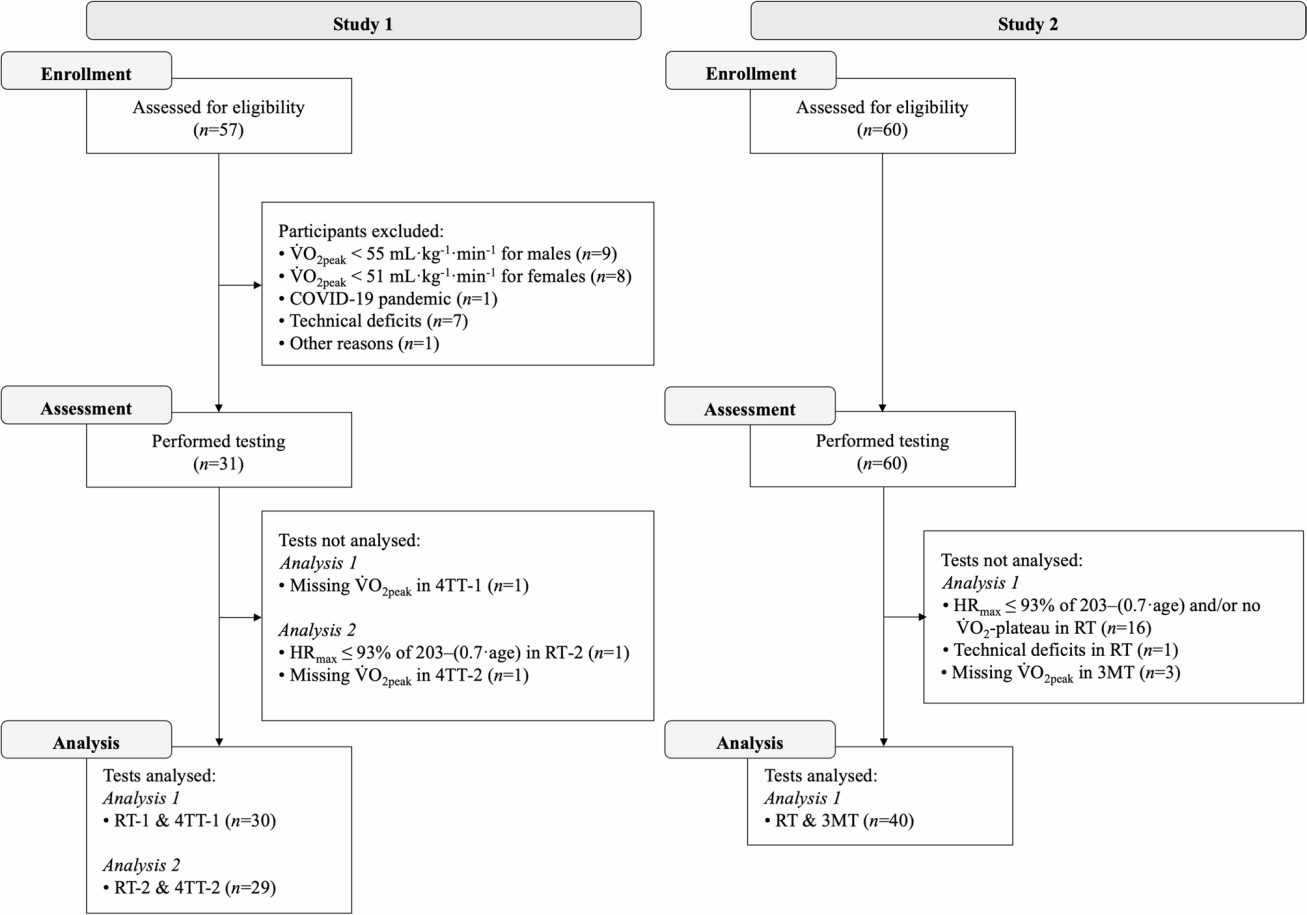
Flow diagram showing inclusion and exclusion of participants in both studies. ${\dot{\text{V}}}$O_2peak_, highest oxygen uptake; RT-1, first ramp test; 4TT-1, first 4-min self-paced time trial; RT-2, second ramp test; 4TT-2, second 4-min self-paced time trial; HR_max_, highest heart rate; RT, ramp test; 3MT, 3-min all-out test.

Both studies enrolled physically healthy individuals between 18 and 39 years of age. Recreational cyclists were defined as having a body mass index ≤ 27 kg·m^-^^2^ and ${\dot{\text{V}}}$O_2peak_ of ≥ 95th percentile (i.e., ≥ 55 mL·kg^−1^·min^−1^ for males and  ≥ 51 mL·kg^−1^·min^−1^ for females) from the American College of Sports Medicine references^23^. CrossFit athletes were defined as those ranked in the top 5% at the 2019, 2020, 2021 or 2022 CrossFit Opens and at the time of testing, training for the next competitive season. Exclusion criteria for both studies were uncontrolled hypertension (systolic blood pressure > 160 mmHg; diastolic blood pressure > 100 mmHg), cardiovascular diseases, febrile infections within the past 14 days, type 1 and type 2 diabetes mellitus, and participation in a clinical trial within the past 4 weeks. Recruitment was carried out through targeted outreach. Recreational cyclists were primarily recruited from a pool of sports science students at the Department of Sport, Exercise and Health at the University of Basel who met the predefined eligibility criteria. CrossFit athletes were recruited through direct inquiries to CrossFit boxes, outreach to high-caliber athletes, and collaboration with the scientific social media platform 'WODSience,' ensuring that only individuals with documented competitive experience were enrolled. The detailed recruitment procedure and the reasons for inclusion and exclusion are shown in Fig. 1.

### Acquisition of participant characteristics

While participants in both studies completed the Physical Activity Readiness Questionnaire, an additional 12-channel resting electrocardiograph was recorded and reviewed by a physician in study 1. Participants responding ‘yes’ to any of the questions on the Physical Activity Readiness Questionnaire (study 1 and 2) and/or showing abnormalities in the electrocardiograph (only study 1) were additionally examined by a physician. Participants’ body height was determined to the nearest 0.5 cm. Further, body mass and body fat content were estimated using a four-segment bioelectrical impedance analysis (Inbody 720; Inbody Co. Ltd., Seoul, South Korea).

### CPET

During the first and third appointment in study 1 and during the only appointment in study 2, a CPET using a ramp protocol was performed on a cycle ergometer (Sport Excalibur; Lode Medical Technology, Groningen, The Netherlands). Participants were allowed to choose their pedaling cadence as long as it was maintained above 60 rpm. After a 3-min warm-up at 50 W for males and females (study 1) or a 5-min warm-up at 100 W for males and 50 W for females (study 2), the workload increased linearly with 30 W per min for all study participants until maximal voluntary exhaustion was reached. The protocol was chosen to achieve a ramp duration of between seven and 18 min^24^. Gas exchange was continuously measured breath by breath throughout the ramp test (MetaMax 3B; Cortex Biophysik GmbH, Leipzig, Germany). Data were averaged over 10-s intervals. Ramp ${\dot{\text{V}}}$O_2peak_ was defined as the highest continuous 30-s value of ${\dot{\text{V}}}$O_2_. Maximal respiratory exchange ratio was defined as the highest value over 10 s measured during the ramp test. In addition to gas exchange measurement, heart rate was also measured continuously using a 12-lead electrocardiograph (Custo med GmbH, Ottobrunn, Germany). Peak heart rate was defined as the highest heart rate value recorded. Peak power output was defined as the highest workload achieved at the point of voluntary exhaustion, while time to exhaustion was defined as the total duration from the start of the ramp phase until maximal voluntary exhaustion. Participants’ rating of perceived exertion was assessed according to Borg scale 6–20^25^. Prior to each study appointment, volume and two-point gas concentration calibration were conducted on the respective metabolic cart.

An increase in ${\dot{\text{V}}}$O_2_ < 50% during the last 50 W of the ramp test relative to the individual increase in the submaximal intensity range was defined as a ${\dot{\text{V}}}$O_2_ plateau. For this objective, the slope of the ${\dot{\text{V}}}$O_2_-work rate relationship of the final 50 W and the submaximal intensity domain (from 80 W to peak power output-60 W) was calculated using linear regression analyses. This definition allows the diagnosis of a ${\dot{\text{V}}}$O_2_ plateau with a risk of false plateau diagnoses of less than 5%^26^. The secondary ${\dot{\text{V}}}$O_2peak_ criterion heart rate was also analysed to verify ${\dot{\text{V}}}$O_2peak_ in the ramp test. It was defined as maximal heart rate ≥ 93% of 208-(0.7·age)^27,28^.

### 4TT and 3MT

In study 1, a 4TT on a cycle ergometer was performed at the second and fourth laboratory appointments. A 3-min warm-up at 50 W was followed by a 10-min submaximal exercise phase at an intensity of 90% of the individual’s first ventilatory threshold determined during the first ramp test. Subsequently, a 3-min recovery phase at 50 W was performed, followed by a 4TT. Participants were instructed to 'cycle as extensively as possible’ and to produce a maximum power output within the 4-min. The workload and pedaling cadence were set at 80% of the ${\dot{\text{V}}}$O_2peak_ determined during the initial CPET. To enable participants to maintain their preferred pedaling cadence, the workload increased quadratically (factor α) in proportion to the increasing pedaling cadence, as outlined by the formula: Power = α (Cadence)^2^. Mean power output during the 4TT was recorded continuously via the cycle ergometer and calculated as the average power output over the four-minute period. While the 4TT in study 1 was performed after at least 24 h of recovery from the ramp test, the 3MT in study 2 was conducted subsequently to a 20-min recovery (5 min active recovery at 30 W + 15 min passive recovery) after the ramp test. The 3MT tests protocol was initiated using 3-min unloaded cycling. The participants were instructed to increase their cadence to approximately 110–120 rpm during the last 10 s of the 3-min unloaded cycling. The unloaded phase was followed by 3MT, in which participants were required to rapidly increase their cadence with the intention of 'cycling as fast as possible’. To induce maximal exhaustion, participants were instructed to maintain their cadence throughout the 3MT as high as possible. The workload (factor α) during the 3MT was set using the linear mode of the ergometer. When participants reached their preferred pedaling (linear factor = workload/cadence), the workload was aligned midway between ${\dot{\text{V}}}$O_2peak_ and the gas exchange threshold, according to the formula: α = Workload at 50% between gas exchange threshold and ${\dot{\text{V}}}$O_2peak_/(cadence^2^ at 50% between the first ventilatory threshold and ${\dot{\text{V}}}$O_2peak_). The described procedure of the 3MT is well established and reported in detail in Constantini et al.^16^.

For both the 4TT and 3MT, the same devices were used as for the ramp tests. 4TT ${\dot{\text{V}}}$O_2peak_ and 3MT ${\dot{\text{V}}}$O_2peak_ were defined as the highest continuous 30-s value of ${\dot{\text{V}}}$O_2_ in the 4TT and 3MT, respectively. Participants’ rating of perceived exertion was assessed according to Borg scale 6–20^25^.

### Data analyses

Descriptive statistics were applied to present participant characteristics and results of the ramp tests, 4TT and 3MT. The primary outcome was the ${\dot{\text{V}}}$O_2peak_ achieved during a ramp test compared to the ${\dot{\text{V}}}$O_2peak_ achieved during either the 4TT or the 3MT. Bland–Altman analyses were used to evaluate the level of agreement between the two measurements^20^. Therefore, the difference between ramp ${\dot{\text{V}}}$O_2peak_ and 4TT ${\dot{\text{V}}}$O_2peak_ respectively between ramp ${\dot{\text{V}}}$O_2peak_ and 3MT ${\dot{\text{V}}}$O_2peak_ were plotted against the mean of both measurements. Only participants achieving a ${\dot{\text{V}}}$O_2_ plateau^29^ or attaining a maximal heart rate ≥ 93% of 208-(0.7·age)^28^ in the ramp test were included in the descriptive statistics and analyses.

A 95% central tolerance interval with a confidence level of 80% was calculated^30^. We used Q-Q plots to assess whether the differences were compatible with a normal distribution. While the differences for study 2 showed some deviations from a normal distribution, the deviations were mostly within the sampling error for a sample size of *n* = 40 (Fig. S1, Supplementary file, Materials and methods, Q-Q-Plots of the differences). Assuming a normal distribution of the differences, the tolerance intervals will contain a minimum of 95% of the central differences in 80% of the cases^30^. To ensure that the measurement range was sufficiently wide for the Bland–Altman analyses, we applied the Preiss–Fisher procedure with 50,000 resamples^22^. According to these analyses, the measurement range was sufficiently wide for all comparisons (Fig. S2, Supplementary file, Materials and methods, Preiss-Fisher procedure). Acceptable range was defined a priori, as ± 0.13 L·min^−1^ (± 3.3%). This value derived from the day-to-day variation in ${\dot{\text{V}}}$O_2peak_ measured in a previous study^31^. Additionally, for each comparison, Lin’s concordance correlation coefficient was calculated^32^. The subsequent standard values were used to estimate the strength of the correlation: < 0.5: weak, 0.5–0.75: moderate, 0.75–0.9: good, > 0.9: excellent^33^. To assess the reliability of the ramp test and 4TT, estimation plots and paired mean differences with a 95% confidence interval (95% CI) were calculated. This was done for both the ${\dot{\text{V}}}$O_2peak_ achieved during the first and second ramp tests and the ${\dot{\text{V}}}$O_2peak_ achieved during the first and second 4TT. We additionally used paired *t*-tests to compare mean ramp ${\dot{\text{V}}}$O_2peak_ and mean 4TT ${\dot{\text{V}}}$O_2peak_ respectively mean 3MT ${\dot{\text{V}}}$O_2peak_. Descriptive data are presented as mean and standard deviation. A significance level of 0.05 was used. Statistical analyses and figures were done in R version 4.1.2^34^. No prior sample size calculations were conducted due to insufficient preliminary data. Nevertheless, the sample size used is considerably larger than most exercise studies comparing ${\dot{\text{V}}}$O_2peak_ across protocols.

## Results

### Participant characteristics

Data from 18 males and 13 females from study 1 and 23 males and 17 females from study 2 were included in the present study (Fig. 1). Participant characteristics from the medical examination and CPET are presented in Table 1.

**Table 1:** Participant characteristics.

Characteristics	Study 1^#^		Study 2	
	Males *n* = 17	Females *n* = 13	Males *n* = 23	Females *n* = 17
Age (years)	23 ± 2 [21–28]	22 ± 2 [19–25]	30 ± 4 [24–39]	29 ± 4 [22–33]
Height (cm)	177 ± 5 [164–183]	165 ± 5 [157–179]	177 ± 6 [166–187]	166 ± 6 [158–175]
Body mass (kg)	70.5 ± 4.3 [63.1–79.6]	59.8 ± 7.8 [49.7–73.7]	87.0 ± 6.7 [73.1–101.7]	65.9 ± 5.0 [58.9–74.8]
Body fat content (%)	11.6 ± 4.0 [3.7–20.5]	21.0 ± 4.5 [15.7–27.7]	9.4 ± 3.1 [3.1–14.9]	9.9 ± 4.4 [3.4–18.7]
${\dot{\text{V}}}$O_2peak_ (mL·kg^−1^·min^−1^)	64.0 ± 5.7 [56.4–74.0]	55.6 ± 5.7 [51.4–64.8]	52.7 ± 3.5 [44.5–59.0]	49.4 ± 3.3 [44.1–58.3]

Data are mean ± standard deviation [range].

${\dot{\text{V}}}$O_2peak_, highest oxygen uptake.

^#^Data included only within the comparison of the first ramp test with the first 4-min self-paced time trial.

### Test–retest reliability of ${\dot{\text{V}}}$O_2peak_ measurement during ramp tests and 4TT

**Fig. 2:**
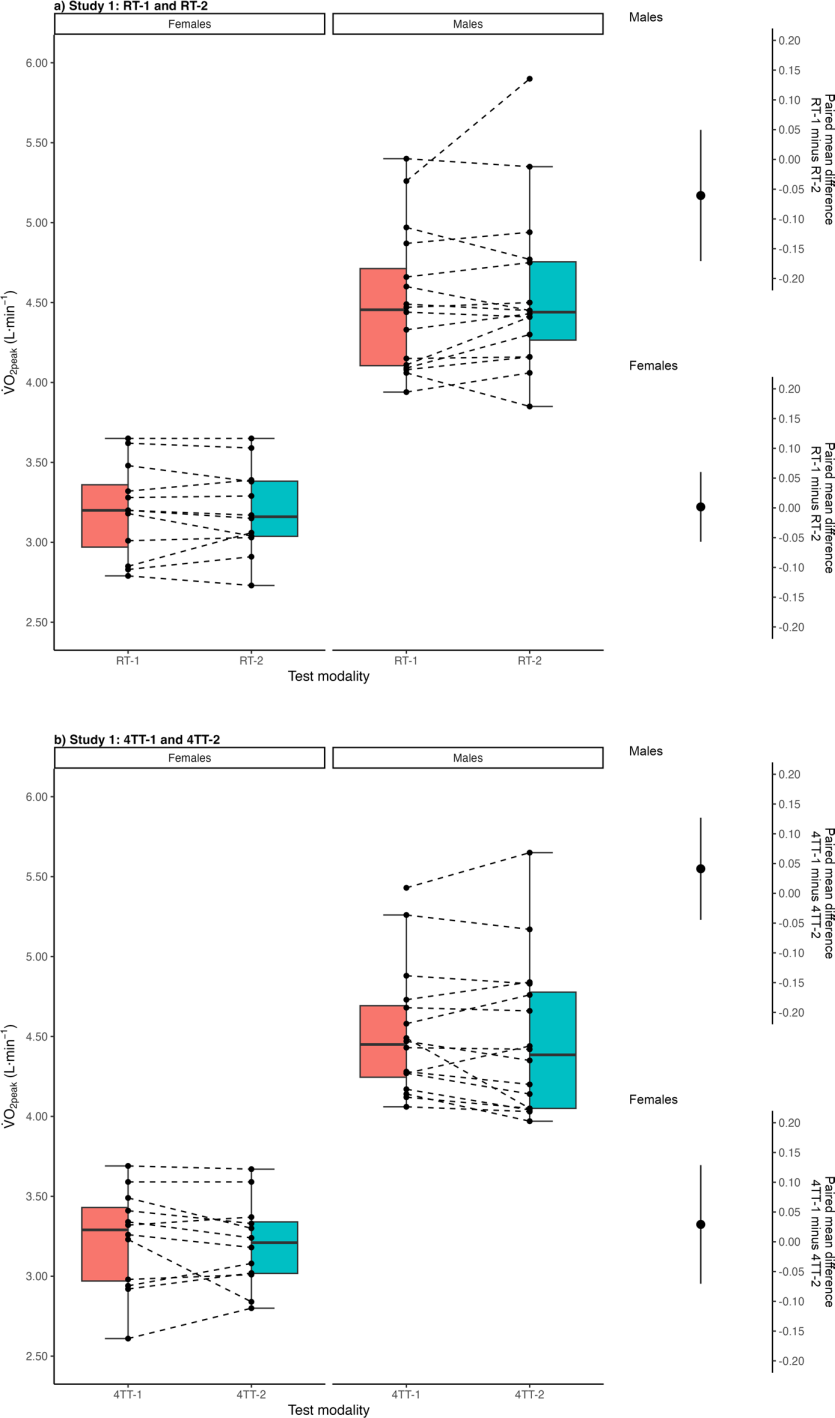
Study 1, Estimation plot for the difference between the highest oxygen uptake (${\dot{\text{V}}}$O_2peak_) achieved during the first ramp test (RT-1) and ${\dot{\text{V}}}$O_2peak_ achieved during the second ramp test (RT-2) (**a**) respectively the ${\dot{\text{V}}}$O_2peak_ achieved during the first 4-min self-paced time trial (4TT-1) and ${\dot{\text{V}}}$O_2peak_ achieved during the second 4-min self-paced time trial (4TT-2) (**b**).

Figure 2 demonstrates the reliability of ${\dot{\text{V}}}$O_2peak_ measured during the first and second ramp tests and ${\dot{\text{V}}}$O_2peak_ measured during the first and second 4TT. For the ramp tests, the paired mean difference was − 0.06 L·min^−1^ (95% CI − 0.17–0.05) for males and 0.002 L·min^−1^ (95% CI − 0.06–0.06) for females. For the 4TT, the respective value was 0.04 L·min^−1^ (95% CI − 0.05–0.13) for males and 0.03 L·min^−1^ (95% CI − 0.07–0.13).

### Comparison of ${\dot{\text{V}}}$O_2peak_ measurement during ramp test, 4TT and 3MT

**Fig. 3:**
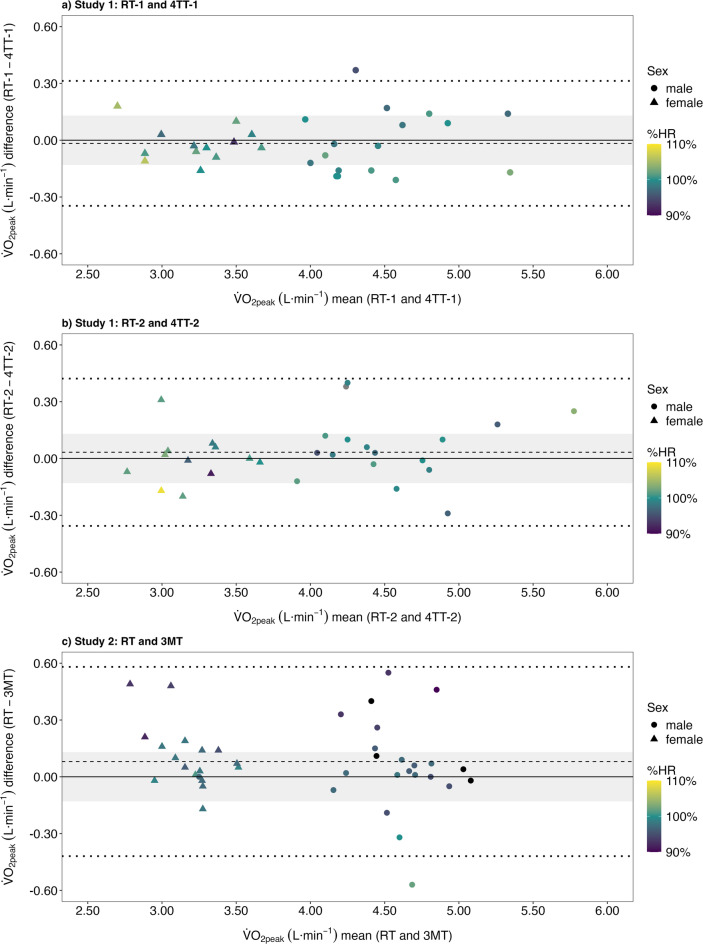
Study 1, Bland–Altman plot of the highest oxygen uptake (${\dot{\text{V}}}$O_2peak_) achieved during the first ramp test (RT-1) and ${\dot{\text{V}}}$O_2peak_ during the first 4-min self-paced time trial (4TT-1) (**a**) respectively during the second ramp test (RT-2) and second 4-min self-paced time trial (4TT-2) (**b**). Study 2, Bland–Altman plot of the ${\dot{\text{V}}}$O_2peak_ achieved during the ramp test (RT) and ${\dot{\text{V}}}$O_2peak_ achieved during the 3-min all-out test (3MT) (**c**). The dashed lines denote the bias (i.e. the mean difference) and the dotted lines denote the 95% central tolerance intervals with an 80% confidence level. The grey shaded area indicates the a priori defined acceptable range of ± 0.13 L·min^−1^ (± 3.3%). The circles represent male participants and the triangles represent female participants. The colour gradient illustrates the individual percentage of the maximum heart rate achieved in the time trial/all-out test compared to the maximum heart rate achieved in the ramp test (reference value).

Table 2 shows the descriptive data for the ramp tests, 4TT and 3MT. Agreements between the two measurement methods for both studies are shown by Bland–Altman plots (Fig. 3). The tolerance limit for the two comparisons in study 1 were − 0.35–0.31 L·min^−1^ (Fig. 3a) and − 0.36–0.42 L·min^−1^ (Fig. 3b), and for the comparison in study 2 − 0.42–0.58 L·min^−1^ (Fig. 3c). In study 1, the mean difference (standard deviation) for the comparison of the first ramp test with the first 4TT and the comparison of the second ramp test with the second 4TT were 0.02 (0.14) L·min^−1^ (*p* = 0.930) and 0.03 (0.16) L·min^−1^ (*p* = 0.873), respectively. In study 2, the mean difference (standard deviation) for the comparison of the ramp test with the 3MT was 0.08 (0.21) L·min^−1^ (*p* = 0.637) (Table S1, Supplementary file, Results, Comparison of the performed exercise tests). No systematic differences in ${\dot{\text{V}}}$O_₂peak_ agreement were observed between male and female participants across both studies (Fig. 3).

**Table 2:** Descriptive data of participants reaching maximum exhaustion during the respective ramp test.

	Study 1						Study 2		
	RT-1			RT-2			RT		
	Total	Males	Females	Total	Males	Females	Total	Males	Females
	n = 30	n = 17	n = 13	n = 29	n = 17	n = 12	n = 40	n = 23	n = 17
Performance									
PPO (W)	359 ± 55	399 ± 33	307 ± 26	366 ± 60	409 ± 35	305 ± 24	373 ± 63	419 ± 39	310 ± 22
TTE (min:sec)	11:23 ± 1:50	12:43 ± 1:07	9:39 ± 0:52	11:39 ± 2:00	13:05 ± 1:09	9:37 ± 0:48	12:50 ± 1:27	13:40 ± 1:18	11:43 ± 0:44
${\dot{\text{V}}}$ O_2peak_ (L·min^−1^)	3.93 ± 0.73	4.47 ± 0.44	3.23 ± 0.30	4.00 ± 0.80	4.57 ± 0.50	3.20 ± 0.27	4.01 ± 0.74	4.58 ± 0.39	3.23 ± 0.17
HR_peak_ (bpm)	188.3 ± 8.3	190.2 ± 8.8	185.9 ± 7.3	188.2 ± 8.1	190.2 ± 8.5	185.3 ± 7.0	182.5 ± 7.4	183.3 ± 6.3	181.5 ± 8.7
RER_peak_	1.21 ± 0.06	1.21 ± 0.05	1.21 ± 0.07	1.19 ± 0.05	1.19 ± 0.04	1.20 ± 0.05	1.14 ± 0.04	1.14 ± 0.04	1.14 ± 0.04
RPE_peak_	19.8 ± 0.5	19.9 ± 0.2	19.6 ± 0.7	19.9 ± 0.4	19.8 ± 0.4	19.9 ± 0.3	19.4 ± 0.7	19.6 ± 0.6	19.1 ± 0.9
${\dot{\text{V}}}$O_2_ plateau and exhaustion criteria									
∆${\dot{\text{V}}}$O_2_ < 50% (n [%])	14 [46.7]	8 [47.1]	6 [46.2]	17 [58.6]	9 [52.9]	8 [66.7]	17 [42.5]	10 [43.5]	7 [41.2]
HR_peak_ ≥ 93% of 208-(0.7·age) (n [%])	28 [93.3]	17 [100]	11 [84.6]	27 [93.1]	17 [100]	10 [83.3]	24 [66.7]	14 [60.9]	10 [58.8]

	4TT-1			4TT-2			3MT		
	Total	Males	Females	Total	Males	Females	Total	Males	Females
	n = 30	n = 17	n = 13	n = 29	n = 17	n = 12	n = 40	n = 23	n = 17
Performance									
P_mean_ (W)	317 ± 58	346 ± 42	279 ± 54	330 ± 61	360 ± 44	287 ± 56	n.r	n.r	n.r
${\dot{\text{V}}}$O_2peak_ (L·min^−1^)	3.95 ± 0.72	4.48 ± 0.42	3.25 ± 0.31	3.97 ± 0.77	4.51 ± 0.49	3.20 ± 0.27	3.93 ± 0.78	4.52 ± 0.40	3.16 ± 0.26
HR_peak_ (bpm)	187.8 ± 7.8	188.9 ± 8.5	186.4 ± 6.8	188.2 ± 8.1	186.0 ± 14.8	185.8 ± 7.3	175.5 ± 7.7^#^	175.1 ± 7.4	175.9 ± 8.3
RPE_peak_	19.7 ± 0.5	19.6 ± 0.6	19.8 ± 0.4	19.8 ± 0.4	19.8 ± 0.4	19.7 ± 0.5	19.9 ± 0.4	19.9 ± 0.4	19.9 ± 0.3

Data are mean ± standard deviation or total numbers [percentage].

RT-1, first ramp test in study 1; RT-2, second ramp test in study 1; RT, ramp test; PPO, peak power output; TTE, time to exhaustion; ${\dot{\text{V}}}$O_2peak,_ highest oxygen uptake; HR_peak_, highest heart rate; RER_peak_, highest respiratory exchange ratio; RPE_peak_, highest rate of perceived exertion; ∆${\dot{\text{V}}}$O_2_, difference between the final and second-to-final 30 W; 4TT-1, first 4-min self-paced time trial; 4TT-2, second 4-min self-paced time trial; 3MT, 3-min all-out test; P_mean_, mean power output.

Note that ∆${\dot{\text{V}}}$O_2_ < 50% of the corresponding increase in ${\dot{\text{V}}}$O_2_ in the submaximal intensity domain indicates the occurrence of a ${\dot{\text{V}}}$O_2_ plateau.

^#^n = 36 due to technical measurement deficits.

In study 1, Lin’s concordance correlation coefficient for ${\dot{\text{V}}}$O_2peak_ was 0.98 (95% CI 0.96–0.99) for both the comparison of the first ramp test with the first 4TT and the comparison of the second ramp test with the second 4TT. In study 2, the respective value for the comparison between the ramp test and the 3MT was 0.95 (95% CI 0.92–0.97). Additionally, ramp ${\dot{\text{V}}}$O_2peak_ and 4TT ${\dot{\text{V}}}$O_2peak_ values respectively ramp ${\dot{\text{V}}}$O_2peak_ and 3MT ${\dot{\text{V}}}$O_2peak_ values were further demonstrated on an individual level in Fig. S3 (Supplementary file, Results, Estimations plots).

## Discussion

The main finding of this comparative study is that a 4TT or 3MT are not directly interchangeable with a ramp test for determining ${\dot{\text{V}}}$O_2peak_ in recreational cyclists and CrossFit athletes. Accordingly, the calculated tolerance limits exceed the a priori defined acceptable range of ± 0.13 L·min^−1^ (± 3.3%), engendering a degree of uncertainty that limits the direct interchangeability of using the 4TT and 3MT as a substitute for the ramp test. The conducted reliability analysis suggests that the mean paired differences in ${\dot{\text{V}}}$O_2peak_ between the ramp tests were below the expected day-to-day variations in ${\dot{\text{V}}}$O_2peak_, demonstrating that a range of ± 0.13 L·min^−1^ (± 3.3%) is not overly narrow. However, as there is no systematic deviation in ${\dot{\text{V}}}$O_2peak_ measured during the 4TT and 3MT compared to ${\dot{\text{V}}}$O_2peak_ measured during the ramp test, and given the good reliability of the 4TT, these tests remain potentially promising for sport-specific performance assessment.

### Measurement of ${\dot{\text{V}}}$O_2peak_ during a 4TT

Previous studies have documented the agreement between ramp ${\dot{\text{V}}}$O_2peak_ and ${\dot{\text{V}}}$O_2peak_ achieved during a time trial^10,12,13,35^. For example, ${\dot{\text{V}}}$O_2peak_ values obtained during a 1000-m^35^ and 4-km^12^ cycle time trial, a 1-mile running time trial^13^, and a 1000-m time trial on a canoe ergometer^10^ were comparable to ${\dot{\text{V}}}$O_2peak_ values attained during an incremental ramp test. In line with these findings^10,12,13,35^, the present comparative study found also no difference between ramp ${\dot{\text{V}}}$O_2peak_ and 4TT ${\dot{\text{V}}}$O_2peak_ when using a *t*-test (Table S1, Supplementary file, Results, Comparison of the performed exercise tests). Further, we found excellent correlations (r ≥ 0.95) between ramp ${\dot{\text{V}}}$O_2peak_ and 4TT ${\dot{\text{V}}}$O_2peak_. Therefore, our results are consistent with previous studies^10,12,13,35^. However, the decisive difference between our studies and previous studies is that we additionally performed analyses using Bland–Altman plots with a a priori acceptable range. These results highlight that direct interchangeability is not given and ${\dot{\text{V}}}$O_2peak_ values measured during 4TT and 3MT should be interpreted with caution. In contrast to a *t*-test or correlation analyses, Bland–Altman plots are appropriate to evaluate agreement between ramp test and time trial measurements of ${\dot{\text{V}}}$O_2peak_ respectively ${\dot{\text{V}}}$O_2peak_ by visualising systematic bias, random variability, and tolerance limits. Bland–Altman plots provide valuable insight into the magnitude of agreement and interchangeability between different measurement methods, with low bias and small standard deviation indicating clinically acceptable conditions^20^. Although our Bland–Altman analyses (Fig. 3) revealed no evidence of systematic bias between ramp tests and 4TT, considerable deviations in ${\dot{\text{V}}}$O₂_peak_ values were observed at the individual level. This conclusion is derived from the deviations between the 4TT ${\dot{\text{V}}}$O_2peak_ and the ramp ${\dot{\text{V}}}$O_2peak_. The calculated tolerance limits exceed the a priori defined acceptable range relying on the expected day-to-day variation of ${\dot{\text{V}}}$O_2peak_^31^. Our findings illustrate that relying exclusively on correlation and mean comparisons as a means to evaluate agreement can lead to potentially misleading conclusions. Nevertheless, the absence of systematic underestimation of ${\dot{\text{V}}}$O_2peak_ in the 4TT and 3MT (Fig. 3) and the good reliability of the time trial in measuring ${\dot{\text{V}}}$O_2peak_ suggest that these protocols could serve as promising complementary tools for providing an overview of ${\dot{\text{V}}}$O_2peak_.

### Measurement of ${\dot{\text{V}}}$O_2peak_ during a 3MT

A previous study by Burnley et al.^11^ applying mean comparisons and Bland–Altman plots stated that 3MT ${\dot{\text{V}}}$O_2peak_ is comparable to ramp ${\dot{\text{V}}}$O_2peak_ in habitually active individuals and thus, concluded that a 3MT can be used interchangeable to a ramp test to establish ${\dot{\text{V}}}$O_2peak_. This conclusion is at odds with the findings of our comparative study. Differences in the consideration of outcome analyses and study protocol between the study by Burnley et al.^11^ and our study should be mentioned here. In the study by Burnley et al.^11^, although Bland–Altman analyses were also performed, no a priori acceptable range was established, which limits the interpretability of their reported agreement. In addition, Burnely et al.^11^ examined a rather small sample size (*n* = 11), resulting in a rather low power of these comparisons. Furthermore, it is important to note that in the Burnley et al. study^11^, unlike in our study 2, the 3MT and the ramp tests were performed on separate days. However, uncertainty remains regarding the direct impact of different recovery phase durations between two maximum exercise tests on ${\dot{\text{V}}}$O_2_ kinetics. For instance, a study involving trained triathletes performing time trials of two to ten minutes in duration following a ramp test found that a maximum recovery phase of 30 min did not significantly impact estimates of critical power and ${\dot{\text{V}}}$O_2peak_ mean values when compared to a recovery phase longer than 24 h^36^. Conversely, it has been consistently demonstrated that prior high-intensity exercise above the lactate threshold accelerates overall ${\dot{\text{V}}}$O_2_ kinetics and reduces the accumulation of blood lactate concentration during subsequent exercise sessions^37^. Consequently, one would expect a higher chance that ${\dot{\text{V}}}$O_2peak_ will be achieved in subsequent time trials respectively all-out tests. Given the varying training protocols used and the remaining ambiguous influence of the recovery duration between two high-intensity exercise sessions, a definitive conclusion remains elusive.

The main principle of the 3MT protocol in study 2 is based on the study by Constantin et al.^16^, aiming to determine critical power, rate of depletion of a finite energy reserve, and ${\dot{\text{V}}}$O_2peak_ in a single-session testing protocol. This test protocol captures physiological performance variables, which may be used to predict CrossFit competition performance^16^. Our results indicate that if ${\dot{\text{V}}}$O_2peak_ is the primary outcome, a ramp test is required. A subsequent 3MT could be used to additionally measure critical power and rate of depletion of a finite energy reserve (if relevant to the particular sport), but imposes substantial additional burden on the participants. If, however, time and resources are limited and critical power and rate of depletion of a finite energy reserve are of highest relevance, use of the 3MT ${\dot{\text{V}}}$O_2peak_ as approximate of ${\dot{\text{V}}}$O_2peak_ is worth considering. Nevertheless, such an approximation should not comprehensively supplant the direct quantification of ${\dot{\text{V}}}$O_2peak_. Inferential, based on the data presented in this study, no conclusions can be drawn regarding the use of a 3MT alone without a preceding ramp test.

### Underlying mechanisms for discrepancies between ramp ${\dot{\text{V}}}$O_2peak_ and 4TT/3MT ${\dot{\text{V}}}$O_2peak_

Noteworthy, most of the aforementioned studies indicating a time trial or all-out test as a valid exercise protocol for assessing ${\dot{\text{V}}}$O_2peak_ did not determine maximum exhaustion during ramp tests using the achievement of ${\dot{\text{V}}}$O_2_ plateau or secondary ${\dot{\text{V}}}$O_2peak_ criteria. The missing determination of maximum exhaustion might lead to an underestimation of the ${\dot{\text{V}}}$O_2peak_ values and thus to false agreements of ramp ${\dot{\text{V}}}$O_2peak_ and 4TT ${\dot{\text{V}}}$O_2peak_ respectively 3MT ${\dot{\text{V}}}$O_2peak_. In the studies mentioned^10–13,35^, it is not clear whether ${\dot{\text{V}}}$O_2peak_ was obtained during the ramp tests, as only one study^13^ investigated the occurrence of a ${\dot{\text{V}}}$O_2_ plateau.

The occurrence of a ${\dot{\text{V}}}$O_2_ plateau in the severe intensity domain indicates the attainment of maximum oxygen uptake during a ramp test^3,29^. However, the incidence of a ${\dot{\text{V}}}$O_2_ plateau, even among athletic populations, is reported to be no higher than 50%, despite participants performing the test with maximal effort^38,39^. Therefore, it is essential to apply at least secondary exhaustion criteria to ensure the highest potential ${\dot{\text{V}}}$O_2_ value^28^. It is conceivable that in the aforementioned studies^10–13,35^, the ${\dot{\text{V}}}$O_2peak_ values achieved during the time trial were only equal or higher because the values referred to by the authors as ramp ${\dot{\text{V}}}$O_2peak_ were not the highest achieveable due to insufficient exhaustion. Hence, the results need to be interpreted cautiously. Whereas there are defined criteria (${\dot{\text{V}}}$O_2_ plateau and secondary ${\dot{\text{V}}}$O_2peak_ criteria) to estimate maximum physical exhaustion during a ramp test^3,27,29^, there are no standardised criteria to determine exhaustion during a time trial or all-out test. In our analyses, physical exhaustion level during the time trial/all-out test was estimated through the calculation of the ratio between the maximum heart rate achieved during the time trial/all-out test and the maximum heart rate achieved during the ramp test. When using heart rate as a criterion to determine exhaustion, for study 2, a plausible explanation for the discrepancies observed between ramp ${\dot{\text{V}}}$O_2peak_ and 3MT ${\dot{\text{V}}}$O_2peak_ could be attributed to an inadequate exhaustion during the 3MT (Fig. 3c), predicated on the corresponding reduced heart rate (highlighted by the purple color). Considering the nature of the CrossFit workouts involving constantly varying functional movements at high intensity^40^, and the elite status of the participants investigated in this study, the absence of maximum exhaustion in the 3MT is rather unlikely. Therefore, it can be argued that the heart rate may not constitute an optimal indicator for quantifying maximum exhaustion during a 3MT, and that the level of exhaustion may not predominantly underlie the observed discrepancies between ramp ${\dot{\text{V}}}$O_2peak_ and 3MT ${\dot{\text{V}}}$O_2peak_.

Our results suggest that performing a time trial or all-out test does not result in consistent over- or underestimation of ${\dot{\text{V}}}$O_2peak_, as indicated by the mean line being approximately 0 for all three comparisons (Fig. 3). When considering the achieved ramp ${\dot{\text{V}}}$O_2peak_ and 4TT ${\dot{\text{V}}}$O_2peak_ respectively 3MT ${\dot{\text{V}}}$O_2peak_ on an individual level (Fig. S3, Supplementary file, Estimation plots), the occurring discrepancies may be due to various and so far unknown factors. These factors could include variations in pacing ability, self-regulated muscle performance^6^, or variations in underlying physiological factors^41^.

### Practical applications

When ${\dot{\text{V}}}$O_2peak_ assessment is critical for performance evaluation or training prescription, reliance on a 4TT or 3MT alone may yield inaccurate results. Using established methods, such as a CPET using a ramp protocol, is recommended for accurate ${\dot{\text{V}}}$O_2peak_ measurement. Ramp protocols offer the advantage of using heart rate and power output to determine ventilatory thresholds, providing insight into aerobic and anaerobic capacity and allowing the definition of individual training zones. In addition, established criteria ensure that athletes test to exhaustion^3,29,38,39^.

Despite these advantages, time trials and all-out tests remain valuable for benchmarking performance-related parameters such as pacing strategies^18^, critical power and the rate of depletion of finite energy reserves^16,19^. Their practicality for real-world applications, including sport-specific contexts, makes them useful beyond ${\dot{\text{V}}}$O_2peak_ determination. However, ramp protocols lack relevance to typical training or competition scenarios. Given the time constraints in competitive sports, efficient data collection is essential for ranking, monitoring and tailoring training. While CPET with a ramp protocol remains the gold standard for ${\dot{\text{V}}}$O_2peak_ assessment and training zone recommendations, incorporating ${\dot{\text{V}}}$O_2peak_ measurements into sport-specific testing protocols can provide an estimate of ${\dot{\text{V}}}$O_2peak_ and therefore may improve performance assessment.

### Strengths and limitations

Notable strengths of the data demonstrated in this comparative study are: examination of both sexes; a high sample size; use of rigorously defined criteria to verify maximum exhaustion; adherence to standardised conditions and use of identical equipment throughout all exercise testing procedures. Further, to our knowledge, this is among the first studies to analyse and discuss the agreement between ramp test and time trials respectively all-out tests to determine ${\dot{\text{V}}}$O_2peak_ using the Bland–Altman approach with an a priori defined acceptable range. Additionally, the study provides a comprehensive individual-level evaluation of the gathered data, setting it apart from previous research.

The inclusion of only recreational cyclists and CrossFit athletes in the present comparative study has to be acknowledged to limit the generalisability of results. To evaluate whether a 3MT could replace a ramp test, the implemented exercise protocol in study 2 may not be the right choice, since we are unable to conclude whether a 3MT can be used independently of a previously performed ramp test to determine ${\dot{\text{V}}}$O_2peak_. Moreover, in study 2, the differences between ramp ${\dot{\text{V}}}$O_2peak_ and 3MT ${\dot{\text{V}}}$O_2peak_ showed some deviations from a normal distribution indicating that the statistical properties of the calculated tolerance limits may hold only approximately. Further, the generalisability to other exercise modalities, such as running or rowing tests, warrants further investigation.

## Conclusion

Although the 4TT and 3MT do provide limited agreement with a ramp test for determining ${\dot{\text{V}}}$O_2peak_ in recreational cyclists and CrossFit athletes, there are no systematic deviations in the measured ${\dot{\text{V}}}$O_2peak_. Alongside their ability to determine sports-specific performance metrics, these results emphasise the potential value of incorporating oxygen uptake measurements during time trials and/or all-out tests for investigating aspects of performance.

## Electronic supplementary material

Below is the link to the electronic supplementary material.

Supplementary Material 1

## Data Availability

The raw data supporting the conclusions of this article will be made available, without undue reservation, by the corresponding authors, Jonathan Wagner and Raphael Knaier.
