# Supplementary material for: Sport-specific performance assessment with oxygen uptake measurements in time trials and critical power tests
Source: Sci Rep. 2025 Jul 21;15:26398. doi: 10.1038/s41598-025-09900-4 (PMC12280066; doi:10.1038/s41598-025-09900-4)
Supplement: Supplementary file 1 — Supplementary Material 1 [file 41598_2025_9900_MOESM1_ESM.docx]

**Sport-Specific Performance Assessment with Oxygen Uptake Measurements in Time Trials and Critical Power Test**

**MATERIALS AND METHODS**

**
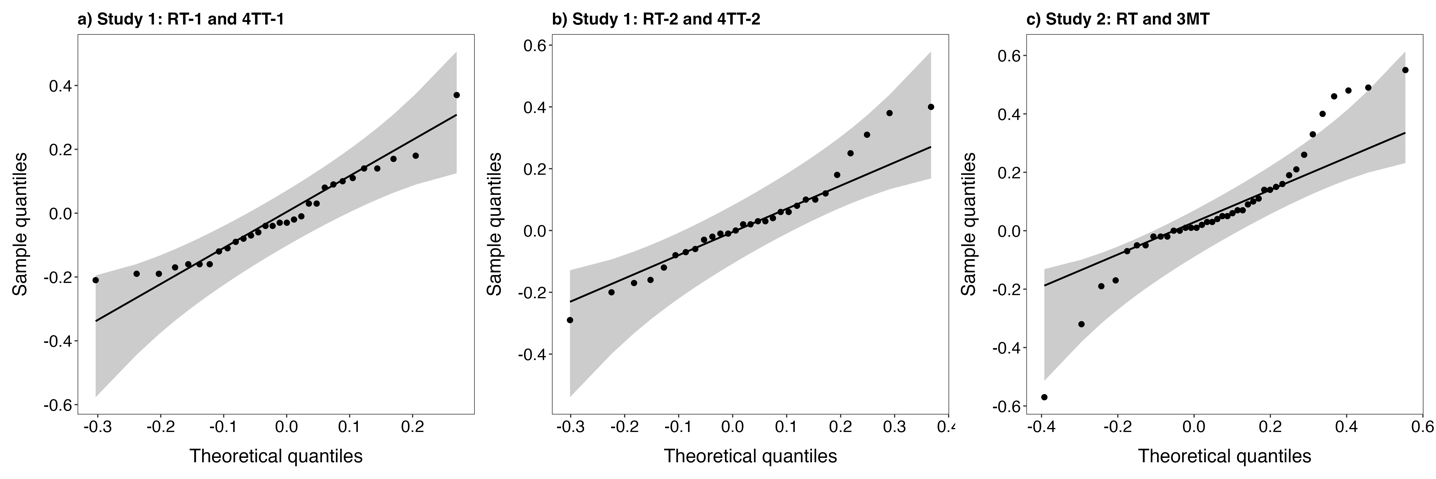
**

**Figure S1.** Q-Q-Plots of the differences. 95% confidence bands are tail sensitive bands^1^.


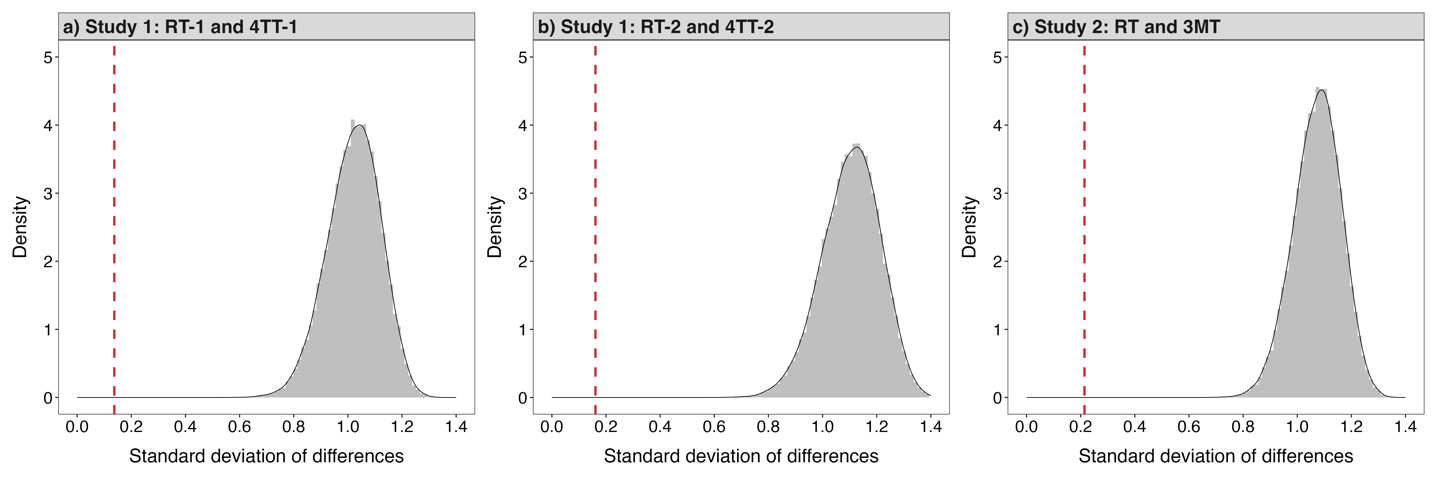


**Figure S2.** Distribution of the standard deviation from 50000 random mispairings according to the Preiss-Fisher procedure. The observed standard deviation is shown as vertical dashed red line. As the observed standard deviation of the difference is clearly smaller than the minimum of the random mispairings indicating that the measurement range is sufficiently wide.

| **RESULTS**  **TABLE S1.** Comparison of the performed exercise tests. | | | | | | | | | | | | | | | |
| --- | --- | --- | --- | --- | --- | --- | --- | --- | --- | --- | --- | --- | --- | --- | --- |
|  | Ramp V̇O_2peak_ (L·min^-1^) | 4TT/3MT V̇O_2peak_ (L·min^-1^) | Difference (L·min^-1^) | *p*-value |  | Ramp V̇O_2peak_ (L·min^-1^) | 4TT/3MT V̇O_2peak_ (L·min^-1^) | Difference (L·min^-1^) | *p*-value |  | Ramp V̇O_2peak_ (L·min^-1^) | 4TT/3MT V̇O_2peak_ (L·min^-1^) | Difference (L·min^-1^) | *p*-value |  |
|  | Total | | | |  | Males | | | |  | Females | | | | |
| **Study 1** | *n* = 30 | | | |  | *n* = 17 | | | |  | *n* = 13 | | | | |
| RT-1 vs.  4TT-1 | 3.93 ± 0.73 | 3.95 ± 0.72 | 0.02 ± 0.14 | 0.930 |  | 4.47 ± 0.44 | 4.48 ± 0.42 | -0.01 ± 0.17 | 0.927 |  | 3.23 ± 0.30 | 3.25 ± 0.31 | -0.02 ± 0.09 | 0.862 |  |
|  | *n* = 29 | | | |  | *n* = 17 | | | |  | *n* = 12 | | | |  |
| RT-2 vs. 4TT-2 | 4.00 ± 0.80 | 3.97 ± 0.77 | 0.03 ± 0.16 | 0.873 |  | 4.57 ± 0.50 | 4.51 (0.49) | 0.06 ± 0.18 | 0.729 |  | 3.20 ± 0.27 | 3.20 ± 0.27 | -0.00 ± 0.13 | 0.976 |  |
| **Study 2** | *n* = 40 | | | |  | *n* = 23 | | | |  | *n* = 17 | | | | |
| RT vs. 3MT | 4.01 ± 0.74 | 3.93 ± 0.78 | 0.08 ± 0.21 | 0.637 |  | 4.58 ± 0.39 | 4.52 ± 0.40 | 0.06 ± 0.24 | 0.611 |  | 3.23 ± 0.17 | 3.16 ± 0.26 | 0.11 ± 0.17 | 0.157 |  |
| Data are mean ± standard deviation.  Ramp V̇O_2peak,_ highest oxygen uptake achieved during the ramp test; 4TT/3MT V̇O_2peak_, highest oxygen uptake achieved during the 4-min self-paced time trial (study 1) respectively 3-min all-out test (study 2); RT-1, first ramp test; 4TT-1, first 4-min self-paced time trial; RT-2, second ramp test; 4TT-2, second 4-min self-paced time trial; RT, ramp test; 3MT, 3-min all-out test. | | | | | | | | | | | | | | | |

**
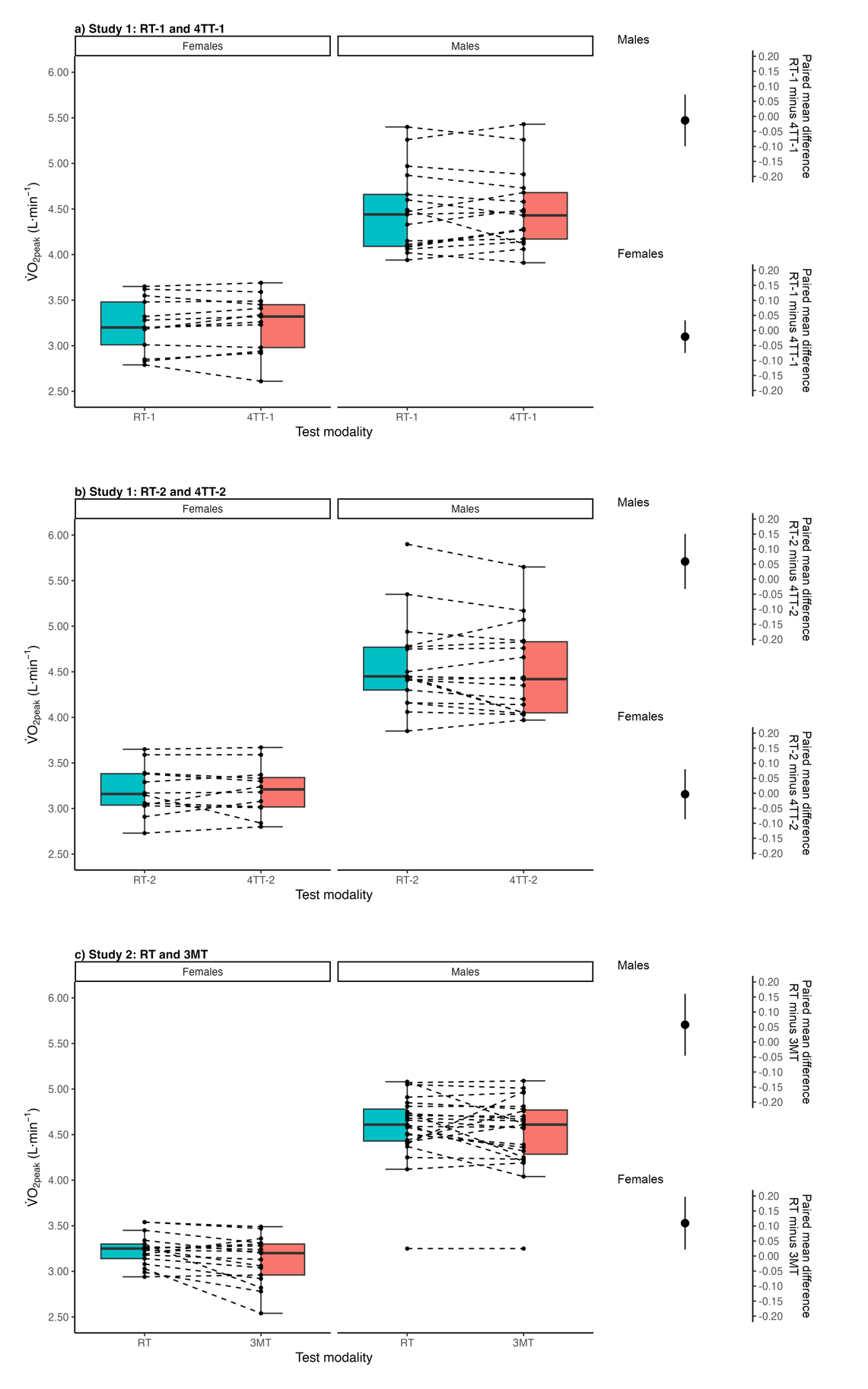
**

**Figure S3.** Study 1, Estimation plot for the difference between the highest oxygen uptake (V̇O_2peak_) achieved during the first ramp test (RT-1) and V̇O_2peak_ achieved during the first 4-min self-paced time trial (4TT-1) (a) respectively during the second ramp test (RT-2) and the second 4-min self-paced time trial (4TT-2) (b). Study 2, Estimation plot for the difference between the V̇O_2peak_ achieved during the ramp test (RT) and V̇O_2peak_ during the 3-min all-out test (3MT). Effect sizes (paired mean differences) and 95% confidence interval are displayed on the right side.

**REFERENCES**

1. Aldor-Noiman S, Brown LD, Buja A, Rolke W, Stine RA. The power to see: a new graphical test of normality. *The American Statistician*. 2013;67(4):249-260. doi:10.1080/00031305.2013.847865
